# Evaluating the Incremental Value of Three-Dimensional Conformer Descriptors for Blood–Brain Barrier Permeability Prediction Using the B3DB Benchmark Dataset

**DOI:** 10.3390/pharmaceutics18081018

**Published:** 2026-08-17

**Authors:** Saurabh Tiwari, Katarzyna Mądra-Gackowska, Marcin Gackowski, Łukasz Szeleszczuk

**Affiliations:** 1 School of Materials Science and Engineering, Yeungnam University, Gyeongsan 38541, Republic of Korea; 2 Department of Geriatrics, L. Rydygier Collegium Medicum in Bydgoszcz, Nicolaus Copernicus University in Torun, 9 Skłodowskiej Curie Str., 85-094 Bydgoszcz, Poland; katarzyna.madra@cm.umk.pl; 3 Department of Toxicology and Bromatology, L. Rydygier Collegium Medicum in Bydgoszcz, Nicolaus Copernicus University in Torun, 2 Jurasza Str., 85-089 Bydgoszcz, Poland; marcin.gackowski@cm.umk.pl; 4 Department of Organic and Physical Chemistry, Medical University of Warsaw, 1 Banacha Str., 02-097 Warsaw, Poland; lukasz.szeleszczuk@wum.edu.pl

**Keywords:** blood–brain barrier, logBB, BBB permeability, molecular descriptors, 3D conformers, machine learning, LightGBM

## Abstract

**Background/Objectives:** Predicting blood–brain barrier (BBB) permeability remains a major challenge in central nervous system (CNS) drug discovery. Three-dimensional (3D) conformer-derived molecular descriptors are often proposed as improvements over conventional two-dimensional (2D) topological representations; however, their incremental predictive value remains unclear. **Methods:** Here, we systematically evaluated six molecular feature sets comprising curated 2D Mordred descriptors, Morgan/ECFP4 fingerprints, 3D conformer-derived descriptors, and their combinations, using LightGBM regression on the B3DB benchmark dataset (1054 compounds with experimental logBB values). The model performance was assessed using 30 independent random splits and 30 Murcko scaffold-based splits. **Results:** The 2D descriptor model achieved mean test R^2^ values of 0.567 ± 0.060 and 0.418 ± 0.085 under random and scaffold splitting, respectively, whereas the 3D descriptor model performed substantially worse (0.430 ± 0.066 and 0.298 ± 0.097, respectively). Incorporating 3D descriptors into the 2D feature set yielded only a marginal improvement under random splitting (+0.008 R^2^; *p* = 0.039), which disappeared during scaffold-based validation (*p* = 0.599). Similarly, adding 3D descriptors to the combined 2D + fingerprint representation did not yield any significant benefits. Fingerprints alone exhibited pronounced scaffold fragility, with the mean R^2^ decreasing from 0.441 to 0.312 between the random and scaffold evaluations. SHAP analysis identified TopoPSA (NO) as the dominant predictor (mean |SHAP| = 0.332), highlighting the central role of desolvation in BBB permeation. Applicability domain analysis showed that 94.8% of the test compounds fell within the structural coverage of the training set. **Conclusions:** Overall, on the B3DB benchmark and under the descriptor implementation evaluated here, 3D conformer descriptors provided limited incremental value and no scaffold-robust advantage over well-curated 2D molecular representations for BBB permeability predictions.

## 1. Introduction

The blood–brain barrier (BBB) is a highly selective physiological interface formed by specialized tight-junction endothelial cells that line the cerebral vasculature. Its primary function is to maintain the homeostatic microenvironment of the central nervous system (CNS) by restricting the paracellular passage of polar, charged, or large molecules, while permitting the transcellular diffusion of small lipophilic compounds. This selectivity constitutes a fundamental challenge and a critical design constraint in CNS drug development. The vast majority of candidate drugs that demonstrate in vitro activity fail to achieve therapeutic concentrations in the brain because they are excluded by BBB efflux transporters or are too hydrophilic to partition into lipid bilayers at a sufficient rate [1].

The computational prediction of BBB permeability, typically expressed as the decadic logarithm of the brain-to-blood concentration ratio (log BB), has become a routine component of early stage drug discovery. The quantitative structure–activity relationship (QSAR) literature on this problem is extensive, spanning linear regression on physicochemical parameters [2], gradient boosting on topological descriptors [3,4], and more recently, graph neural networks (GNNs) operating directly on molecular graphs [5]. A recurring question across these approaches is which molecular representation best captures the BBB-relevant structural features, principally, the balance between lipophilicity and polar desolvation penalty [6]. Two-dimensional topological descriptors, among which topological polar surface area (TPSA), logP surrogates, hydrogen-bond donor and acceptor counts, and ring-system descriptors are the most predictive, have historically dominated BBB QSAR models [2,3]. The theoretical appeal of three-dimensional (3D) conformational descriptors lies in the expectation that they capture the molecular shape, electrostatic distribution, and geometry-dependent polar atom exposure in a manner that 2D graph-based features cannot be achieved. Descriptors such as the Charged Partial Surface Area (CPSA) family [7], 3D-MoRSE autocorrelation codes, plane-of-best-fit (PBF) [6,8], and moments of inertia encode explicit spatial information from energy-minimized geometries. Several studies have incorporated three-dimensional molecular information into BBB permeability models. Adenot and Lahana built classification models distinguishing CNS from non-CNS compounds using physicochemical descriptors that included polar surface area-related features, demonstrating that surface-area characteristics are central to BBB discrimination [9]. Mente and Lombardo evaluated Charged Partial Surface Area (CPSA) descriptors for three-dimensional charge-weighted surface area quantities computed from molecular conformers for log BB regression using recursive partitioning and reported leave-group-out Q^2^ values of 0.51 for the CPSA descriptor set, comparable to simpler physicochemical representations [10]. Despite these efforts, a systematic paired comparison of explicit 3D conformer descriptors against curated 2D representations under both random and scaffold-based validation on a large, standardized experimental dataset has not yet been reported. This motivated the present study.

Despite this theoretical motivation, rigorous head-to-head comparisons of 2D and 3D descriptor sets under split-consistent evaluation protocols are limited. Most published studies focus on maximizing the predictive performance of a single dataset partition rather than isolating the marginal contribution of 3D geometry. This distinction is important in practice because if any improvement from 3D descriptors is observed only under random splitting, where training and test molecules share scaffolds, it cannot be attributed to the genuine learning of transferable geometry-dependent chemical properties. The appropriate test is whether the gains persist under scaffold-based cross-validation, which evaluates generalization to structurally novel compounds. This study directly addressed this issue. Using the B3DB experimental dataset [3], the largest single-source collection of experimental logBB measurements currently available, we conducted a comprehensive evaluation by comparing six molecular feature representations using LightGBM regression across 30 independent random splits and 30 independent Murcko scaffold-based splits. The primary question is whether the 3D conformer descriptors provide a scaffold-robust improvement over the optimized two-dimensional (2D) topology. We also characterized feature importance through SHAP analysis, assessed the structural applicability domain via leverage-based William’s plot analysis, and directly compared the 2D and 3D polar surface area descriptors.

Previous work on the B3DB dataset demonstrated that curated 2D molecular descriptors achieved a test R^2^ = 0.604 using a gradient boosting framework based on a single optimized random partition [11]. The present study differs from this work in three important aspects: (i) it evaluates model performance across 30 independent random and scaffold-based splits, enabling paired statistical comparisons and a more comprehensive assessment of model robustness and generalizability; (ii) it systematically compares six molecular feature-set combinations within a controlled factorial design rather than optimizing a single molecular representation; and (iii) its primary objective is to determine whether explicit 3D conformer geometry provides additional scaffold-robust predictive value beyond conventional 2D topology, an aspect not examined in the previous study. Therefore, the aim of the present study was not to achieve the highest possible R^2^ on the B3DB benchmark but rather to provide a rigorous and reproducible evaluation of the incremental contribution of 3D conformer descriptors under controlled validation conditions.

## 2. Materials and Methods

### 2.1. Dataset

All analyses used the B3DB regression dataset (v1.1.1), a curated collection of experimental log BB measurements assembled from the literature by Meng et al. [3]. The full regression set contained 1058 compounds spanning diverse chemical spaces, with log BB values ranging from −2.69 to +1.70 (mean: −0.08, standard deviation: 0.75). After the removal of four compounds for which RDKit (version 2023.09.5) failed to generate valid three-dimensional conformers (99.6% success rate), the final analysis set comprised 1054 compounds in total. No additional filtering was applied to preserve the chemical diversity of the benchmark. Additional information on the compounds included in the final dataset is presented in Supplementary Table S1.

### 2.2. Molecular Featurization

Three molecular representation strategies were evaluated in this study: The Mordred cheminformatics package (v1.2.0) [12] was used to calculate 608 two-dimensional molecular descriptors for all 1058 compounds after hydrogen suppression. A three-stage selection procedure was applied to obtain a compact and informative feature set. Descriptors exhibiting near-zero variance (SD < 0.01) were removed, followed by Pearson correlation filtering at |r| > 0.95, retaining descriptors exhibiting higher mutual information with logBB within each pair. The remaining descriptors were ranked using a composite score derived from the mutual information with logBB and 100-tree random forest feature importance, yielding a final set of 60 descriptors for the QSAR models. A complete list of the retained descriptors is provided in Supplementary Table S2. Topological polar surface area (TopoPSA (NO)), equivalent to RDKit’s CalcTPSA implementation based on the Ertl method [2], ranked highest according to both selection criteria. In parallel, circular Morgan fingerprints (ECFP4) were generated using RDKit’s GetMorganFingerprintAsBitVect function with radius of 2 and a fingerprint length of 1024 bits [13,14]. This representation encodes the local atomic environments and molecular substructures as binary feature vectors and has been widely applied in molecular property-prediction studies.

To evaluate the contribution of three-dimensional molecular information, conformers were generated using the RDKit ETKDGv3 distance-geometry algorithm followed by MMFF94 force-field energy minimization [15] with randomSeed = 42. A single optimized conformer was retained for each molecule. Mordred was then used to calculate descriptors requiring three-dimensional coordinates, including charge-partial surface area (CPSA) descriptors [7], 3D-MoRSE descriptors, geometrical shape indices, principal moments of inertia, and plane-of-best-fit descriptors [6,8]. Descriptors with more than 20% missing values or zero variance were removed, resulting in a final set of 40 three-dimensional descriptors. The WHIM, GETAWAY, and RDF descriptors were not available in the installed Mordred implementation, and were therefore excluded from the analysis.

### 2.3. Machine Learning Framework

LightGBM [16] was employed as the primary regression algorithm for all repeated analyses, using the hyperparameters n_estimators = 150, learning_rate = 0.05, max_depth = 5, and verbose = −1. The random_state parameter was varied across repetitions. The use of a single learning algorithm throughout the study ensured that all reported means, standard deviations, and paired statistical comparisons reflected differences in molecular representation rather than differences in model selection. The model performance was evaluated using two complementary validation strategies across 30 independent repetitions of each strategy. In the random-split protocol, compounds were randomly divided into training (80%) and test (20%) sets using train_test_split with random_state values ranging from 0 to 29. In the Murcko scaffold-based protocol, compounds were partitioned according to their Bemis–Murcko core scaffolds [17], ensuring that no scaffold was present in both the training and test sets. The final dataset comprised 1054 compounds containing 649 unique Murcko scaffolds (mean: 1.62 compounds per scaffold). Scaffold-based splitting was implemented using GroupShuffleSplit (scikit-learn version 1.3.2) with scaffold SMILES as group labels, targeting an approximate 80:20 training-to-test ratio for each repetition. Scaffold-based splitting was treated as the primary validation criterion throughout this study because it provides a direct assessment of model generalization to structurally novel compounds, the scenario most relevant to prospective virtual screening, where newly designed compounds routinely possess scaffolds absent from historical training data. Under random splitting, compounds with the same core scaffold frequently appear in both the training and test sets, which inflates the performance estimates because the model has been exposed to closely related structural environments during training. In contrast, the scaffold-split test set contains only compounds whose core ring systems were entirely unseen during training, making the scaffold-split performance the appropriate measure of transferable predictive signals. This approach is consistent with the MoleculeNet benchmark convention [5] and the Bemis–Murcko scaffold framework [17].

### 2.4. Statistical Testing

Paired comparisons between feature sets used both the paired Student’s *t*-test and the Wilcoxon signed-rank test (*n* = 30 paired replicates per comparison, scipy.stats (SciPy version 1.11.4) [18]). Four comparisons were pre-specified: (1) 2D vs. 2D + 3D, (2) 2D + FP vs. 2D + FP + 3D, (3) 2D vs. FP-only, and (4) 2D vs. 2D + FP. A two-sided significance threshold of α = 0.05 was applied to all analyses. The scaffold-split paired test was considered the primary evidence for generalizability, since random-split tests include inflated statistical power attributable to scaffold leakage between the training and test sets.

### 2.5. SHAP Analysis

Feature importance was characterized using TreeExplainer from the SHAP library [19], which was applied to a LightGBM model trained on the curated 2D descriptor set using a fixed random split (random_state = 42, 80:20 training-to-test, n_train = 843, n_test = 211). The mean absolute SHAP values were computed over the full test set and used to rank the descriptors according to their global contributions to log BB prediction.

### 2.6. Applicability Domain Assessment

The applicability domain of the developed model was evaluated using the leverage-based approach recommended by the OECD principles for QSAR validation [20] and described by Sahigara et al. [21]. Leverage values were calculated as hi = xi^T^(X^T^X)^−1^xi using the standardized (zero-mean, unit-variance) 60-descriptor training matrix X. The warning leverage threshold was defined as h* = 3(*p* + 1)/*n*, corresponding to 0.217 for the present dataset (*p* = 60; *n* = 843). Model applicability was assessed using a Williams plot, in which the standardized residuals were plotted against leverage values. This approach enables the identification of structurally influential compounds (h > h*) and compounds exhibiting unusually large prediction errors (|residual| > 3σ), thereby distinguishing structural extrapolation from prediction uncertainty.

## 3. Results

### 3.1. Dataset and Conformer Generation

The B3DB regression set spans log BB values from −2.69 to +1.70 (mean −0.08, SD 0.75), covering approved CNS drugs, peripheral drugs, environmental compounds, and reference nonpenetrant structures. ETKDGv3 + MMFF94 conformer generation succeeded for 1054 out of 1058 compounds (99.6%); the four failures were inorganic or near-inorganic structures for which the MMFF94 atom typing was not defined. The 1054-compound set contained 649 unique Murcko scaffolds (mean, 1.62 compounds per scaffold; range, 1–18), confirming substantial structural diversity appropriate for scaffold-based cross-validation.

### 3.2. Feature Set Comparison, 30-Repeat LightGBM Analysis

Table 1 summarizes the mean test R^2^ values obtained from 30 independent random splits and 30 independent Murcko scaffold-based splits for each molecular feature representation. The difference between the random and scaffold performances provides an estimate of the loss in predictive accuracy associated with generalization to previously unseen chemical scaffolds.

**Figure 1:**
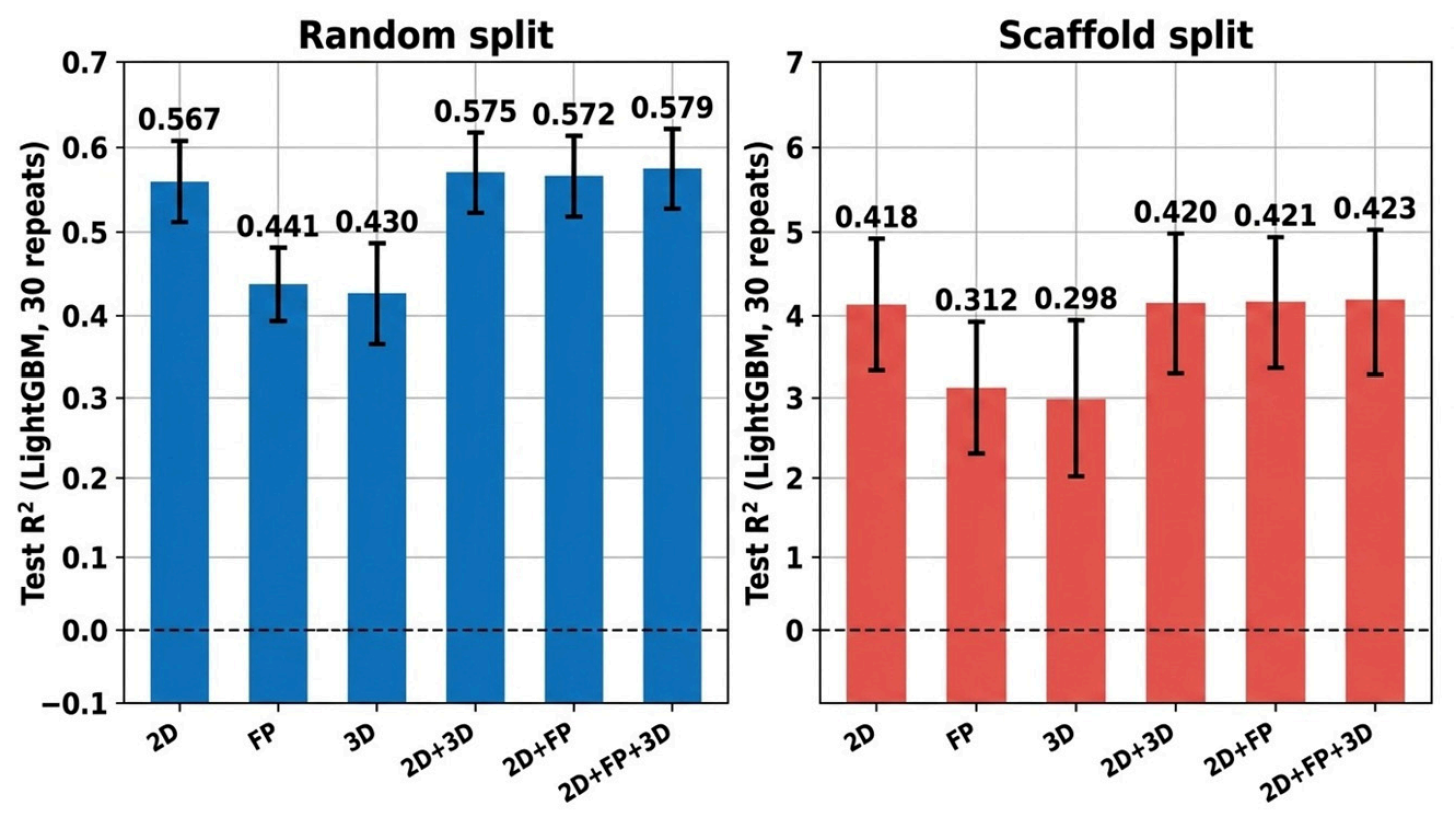
Mean test R^2^ (±1 SD, 30 repeats, LightGBM) across six feature sets under random splitting (**left**, blue) and Murcko scaffold splitting (**right**, salmon). The error bars represent the standard deviations across 30 independent splits.

Figure 1 provides a visual comparison of the predictive performances of the six molecular feature representations under both validation strategies.

The curated 2D descriptor set achieved a mean test R^2^ of 0.567 ± 0.060 (random) and 0.418 ± 0.085 (scaffold), serving as a reference for subsequent comparisons. The 3D descriptor set performed substantially below this baseline at 0.430 ± 0.066 (random) and 0.298 ± 0.097 (scaffold), confirming that 3D geometry alone is less informative than 2D topology for this specific task. The fingerprints showed a scaffold fragility gap of 0.129 R^2^ units (0.441 random vs. 0.312 scaffold), reflecting overfitting to the scaffold-specific substructure patterns that did not transfer to the unseen ring systems. Adding 3D descriptors to the 2D baseline produced a small numerical gain of +0.008 R^2^ units under random splitting (2D + 3D: 0.575 vs. 2D: 0.567) and a negligible +0.003 under scaffold splitting (0.420 vs. 0.418). Similarly, the three-way combination 2D + FP + 3D yielded the numerically highest mean R^2^ across both split types (random: 0.579; scaffold: 0.423), but the gains over the 2D-only baseline were modest (+0.012 random, +0.005 scaffold), and as demonstrated in the following section, statistically indistinguishable from zero in the scaffold-based evaluation. The observed gains were modest and did not remain statistically significant during scaffold-based validation. Consequently, any contribution from the three-dimensional descriptors appears too small to provide a robust improvement in generalization performance. To further characterize the contribution of the 3D descriptor set in isolation, the 3D-only model achieved an R^2^ of 0.430 ± 0.066 under random splitting and 0.298 ± 0.097 under scaffold splitting, which was substantially lower than the corresponding 2D baseline by 0.137 and 0.120 R^2^ units, respectively. Among the retained 40 3D descriptors, the most influential descriptor families were the Charged Partial Surface Area (CPSA) descriptors, including PNSA1, PNSA2, and PNSA3 (partial negative surface area variants), together with 3D-MoRSE autocorrelation descriptors and geometrical shape indices describing molecular moments of inertia and plane-of-best-fit characteristics. Although these descriptors encode explicit conformational information, none provided predictive performance that exceeded that of the established 2D polarity descriptors. At the individual descriptor level, the strongest 3D feature showed a univariate association with log BB comparable to TopoPSA (NO) (r^2^ ≈ 0.29); however, the multivariate 3D descriptor model did not achieve a comparable performance to the 2D representation. These findings suggest that for the present BBB permeability dataset, the geometric information captured by explicit 3D conformer descriptors does not provide substantial additional explanatory power beyond that already encoded by the 2D molecular topology.

### 3.3. Statistical Significance Testing

Table 2 summarizes the paired *t*-test and Wilcoxon signed-rank test results for the four pre-specified comparisons. Scaffold-based validation was considered the primary measure of model generalizability. The complete results of the paired statistical tests are presented in Supplementary Table S4.

Under scaffold splitting, the primary metric, neither the addition of 3D descriptors to 2D (*p* = 0.599, *t*-test; *p* = 0.477, Wilcoxon) nor the addition of 3D descriptors to 2D + FP (*p* = 0.801; *p* = 0.984) was statistically significant. Under random splitting, the 2D vs. 2D + 3D comparison reached borderline significance (*p* = 0.039, *t*-test; *p* = 0.055, Wilcoxon), and the 2D vs. 2D + FP comparison was also borderline (*p* = 0.051, *t*-test; *p* = 0.033, Wilcoxon test). However, both gains disappeared entirely under scaffold splitting (*p* = 0.599 and *p* = 0.290, respectively), indicating that these marginal improvements reflect scaffold co-occurrence between the training and test sets rather than genuinely learnable, geometry-dependent chemistries. This pattern, namely a statistically marginal effect under random splitting and a null effect under scaffold-based validation, is consistent with scaffold-dependent information that does not translate into meaningful out-of-scaffold generalization and therefore cannot be interpreted as evidence of a transferable three-dimensional signal. The comparison of 2D descriptors versus fingerprints-only was highly significant for both split types (random *p* < 0.001; scaffold *p* < 0.001), confirming that topological physicochemical descriptors carry substantially more generalizable predictive information than substructure-encoding binary fingerprints in the BBB regression.

### 3.4. SHAP Feature Importance

**Figure 2:**
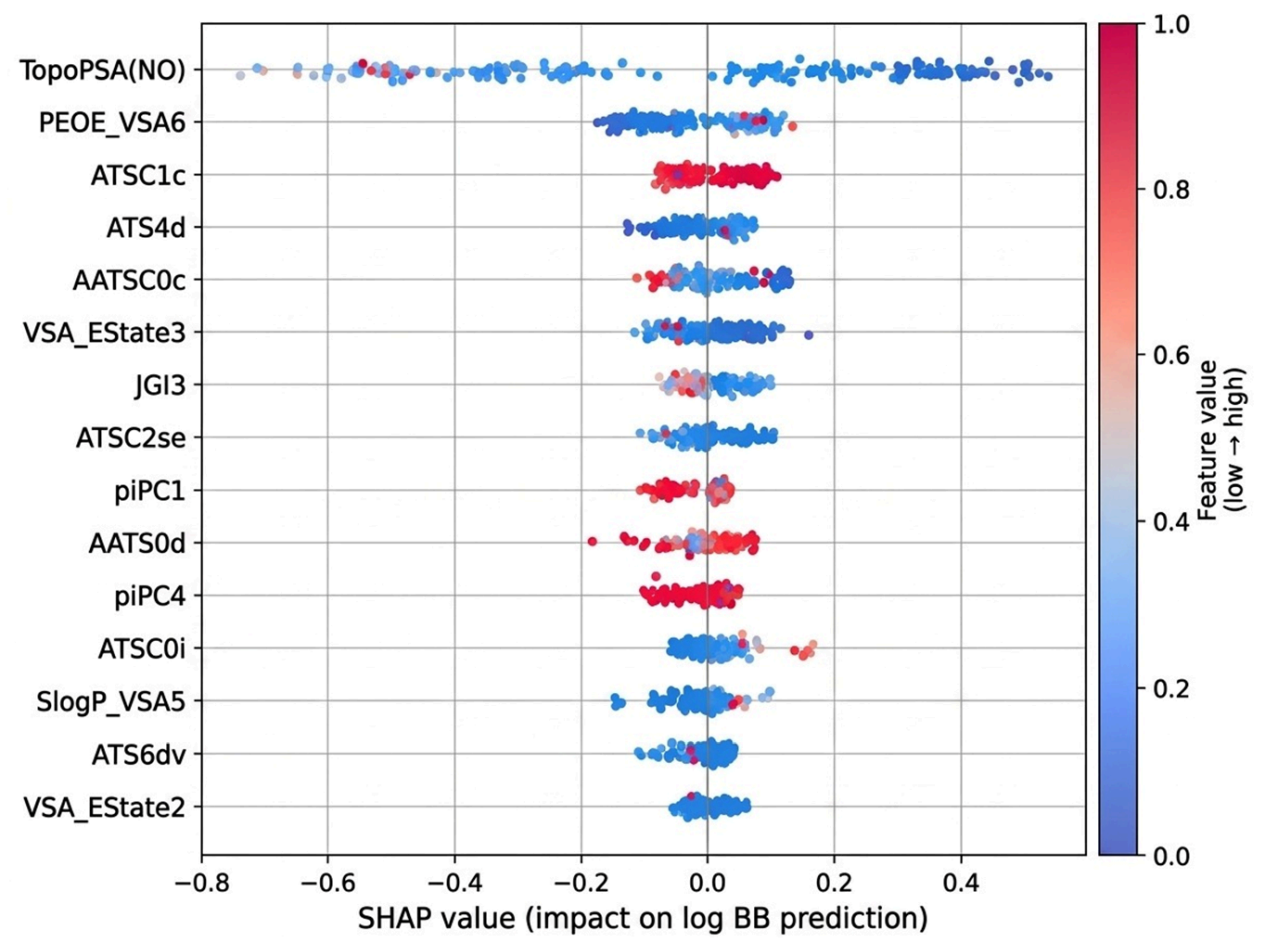
SHAP beeswarm plot for the curated 2D descriptor model (test set, *n* = 211). The color encodes the standardized feature value (red = high and blue = low). The TopoPSA (NO) feature dominated with a mean |SHAP| of 0.332, which was approximately four times that of the second-ranked feature (PEOE_VSA6 = 0.080).

TopoPSA (NO) was the most influential descriptor by a substantial margin (mean |SHAP| = 0.332; Figure 2), consistent with the desolvation mechanism underlying passive BBB permeation. The complete SHAP ranking is presented in Supplementary Table S5. High TPSA values were uniformly associated with negative SHAP values, reflecting the well-established penalty for polar surface area crossing the blood–brain interface.

The second-ranked descriptor, PEOE_VSA6 (mean |SHAP| = 0.080), is a van der Waals surface area bin weighted by Gasteiger partial charges, and the third, ATSC1c (0.049), is a centered Broto–Moreau autocorrelation weighted by atomic charge at lag 1. Both descriptors encode complementary aspects of the molecular electrostatic surface, and together with TPSA, explain the dominant mechanistic gradient. The remaining top features included π-system connectivity indices (piPC1 and piPC4) and an additional charge-weighted surface area. To provide feature-level evidence for the limited contribution of 3D descriptors, an equivalent SHAP analysis was performed on the 3D-only LightGBM model using the same fixed random split as that of the corresponding 2D model interpretation. The highest-ranked 3D descriptors according to mean absolute SHAP value were PNSA1 (partial negative surface area from the CPSA family; mean |SHAP| = 0.098), PNSA2 (0.061), and PNSA3 (0.054). These contributions were considerably smaller than the influence of TopoPSA (NO) in the 2D model (mean |SHAP| = 0.332), highlighting the stronger predictive role of conventional 2D polar surface descriptors in this dataset. Furthermore, when the 2D and 3D descriptors were combined in the 2D + 3D model, no 3D-derived descriptor appeared among the top-15 features ranked by the mean absolute SHAP value. This indicates that the information provided by explicit 3D descriptors largely overlaps with or is already captured by the dominant 2D descriptors when both descriptor classes are available simultaneously.

### 3.5. Two-Dimensional Versus Three-Dimensional Polar Surface Area

**Figure 3:**
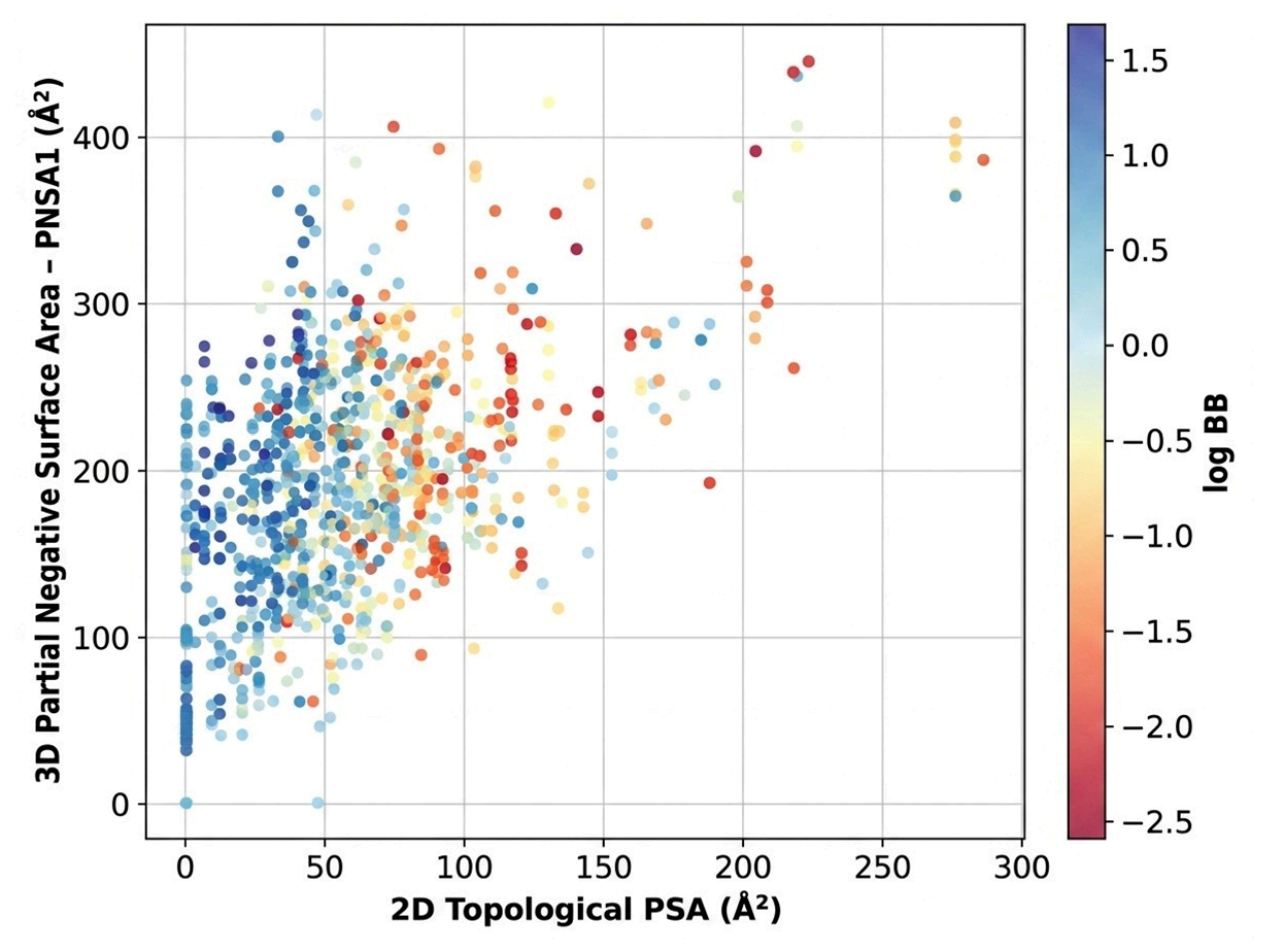
Scatter plot of 2D TopoPSA (NO) versus 3D partial negative surface area descriptor PNSA1 (MMFF94 conformer), color-coded by the experimental log BB. Pearson r = 0.52 (*n* = 1054); high-log BB compounds (blue) cluster toward low PSA values on both axes.

The Pearson correlation between 2D TopoPSA (NO) and 3D PNSA1 (partial negative surface area from the CPSA descriptor family, computed on MMFF94 geometry-optimized conformers) was r = 0.52 (Figure 3). This moderate correlation reflects the expected relationship between the two quantities while confirming that they are not equivalent: the 2D TPSA integrates tabulated increments for polar atoms from the molecular graph, whereas the 3D PNSA1 sums the solvent-accessible surface area contributions of negatively charged atoms from the conformational geometry. Despite this numerical divergence, both descriptors showed comparable univariate predictive power (individual Pearson r^2^ ≈ 0.29).

This result supports the interpretation that the thermodynamic desolvation cost of BBB crossing is adequately captured by the 2D topological approximation, and that the additional geometric details in the 3D PSA do not reduce the residual predictive error beyond that encoded by the 2D topology.

### 3.6. Applicability Domain

**Figure 4:**
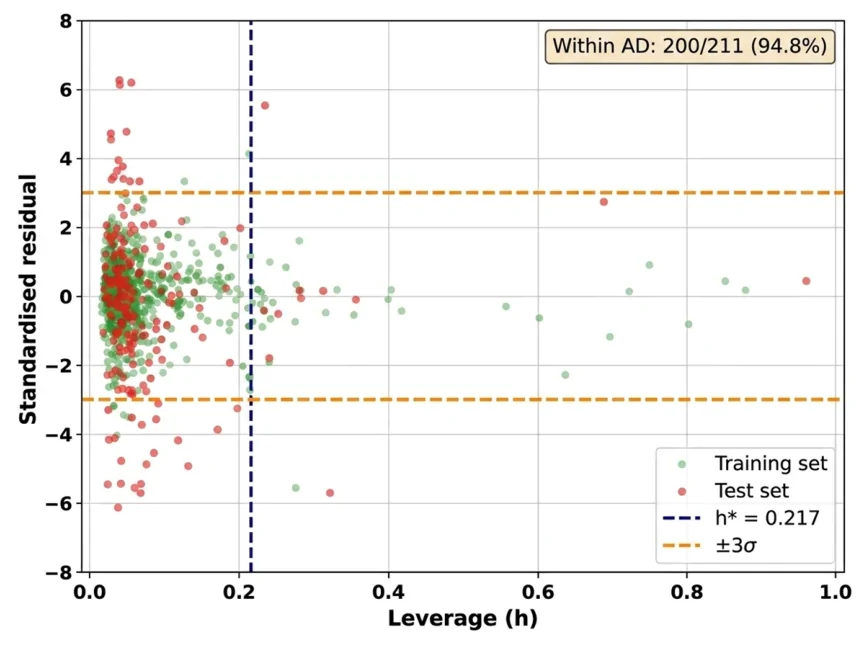
Williams plot for the 2D descriptor LightGBM model (random_state = 42). The vertical dashed line marks h* = 0.217 (*p* = 60, *n* = 843), and the horizontal dashed lines mark ±3σ (where σ is derived from the training residuals). The compound with the highest leverage (h = 0.95) was ribavirin, a nucleoside analog, relative to the predominantly organic drug-like training set used.

Figure 4 presents the Williams plot used to assess the structural applicability domain of the curated 2D descriptor-based model. The warning leverage threshold was h* = 3(61)/843 = 0.217.

Of the 211 test compounds, 200 (94.8%) were within their structural applicability domains (h ≤ h*). The test compound with the largest leverage (h = 0.95) was ribavirin, a ribose-containing nucleoside antiviral that is structurally atypical compared to the predominantly lipophilic heteroaromatic training corpus. Structural AD and large residual flags were reported as independent variables. Of the test compounds, 37/211 (17.5%) showed |residual| > 3σ despite being structurally within the training chemical space, suggesting that they occupy sparse regions or involve active efflux mechanisms not captured by the physicochemical descriptors.

**Figure 5:**
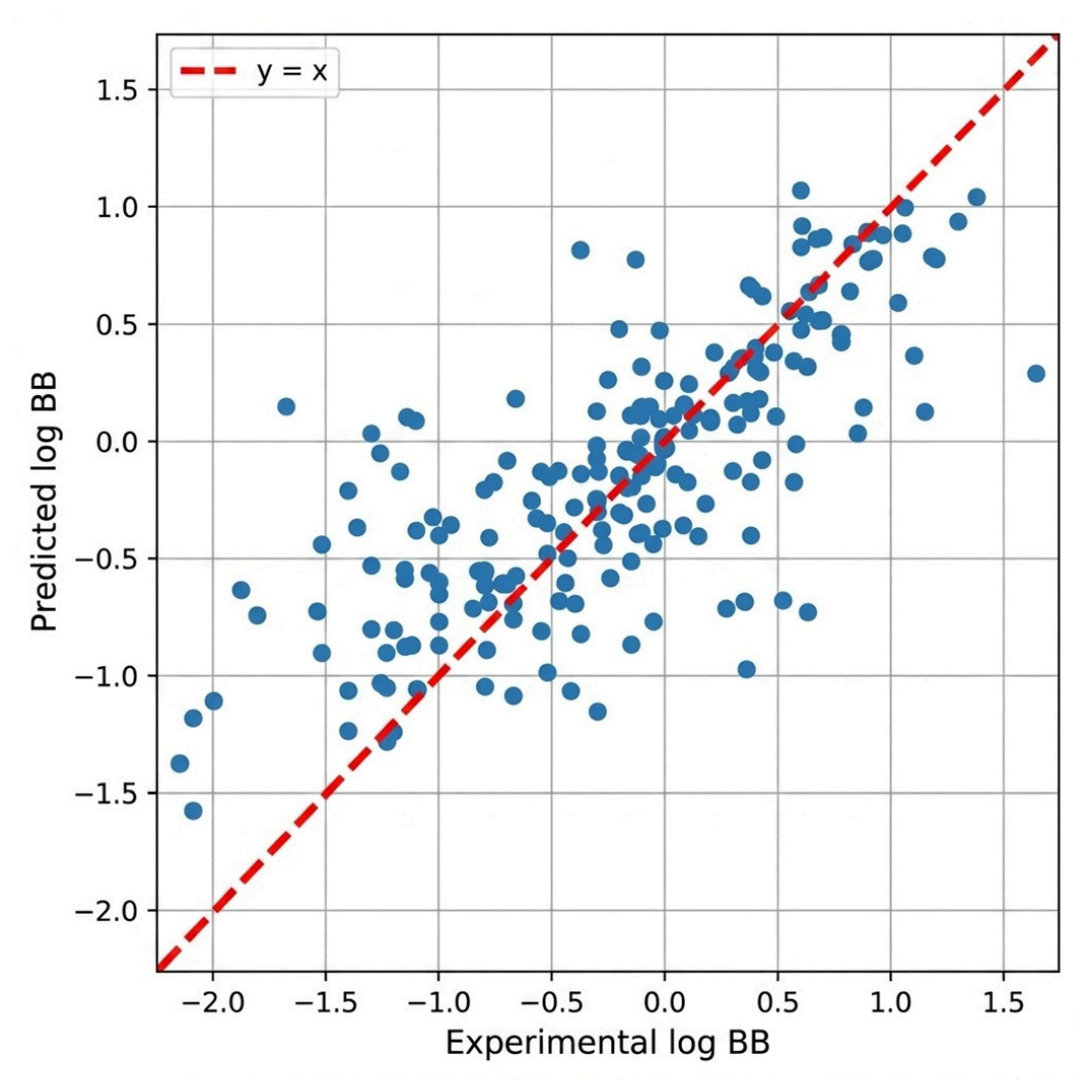
Predicted vs. experimental log BB for the 2D descriptor LightGBM model (test set *n* = 211, random_state = 42). R^2^ = 0.562, RMSE = 0.501. Dashed red line: *y* = *x*.

Figure 5 shows the relationship between the predicted and experimental logBB values for the representative test split (random state = 42). The model achieved R^2^ = 0.562 and RMSE = 0.501, with most compounds clustered near the identity line.

## 4. Discussion

The central finding of this study, evaluated on the B3DB benchmark using Mordred-derived 3D descriptors calculated from single ETKDGv3 + MMFF94 conformers, is that three-dimensional conformer-derived descriptors provide small numerical gains in log BB prediction under random splitting, but these improvements do not persist under scaffold-based validation. Therefore, within the present ligand-based BBB permeability modeling framework, explicit 3D conformational descriptors do not demonstrate consistent transferable predictive value beyond that captured by conventional 2D molecular representations. This distinction is more nuanced than a simple null result. The highest mean R^2^ across all 30-repeat evaluations was observed for the 2D + FP + 3D combination (random: 0.579; scaffold: 0.423), and the 2D vs. 2D + 3D comparison reached borderline significance under random splitting (*p* = 0.039). However, the appropriate interpretation is that a +0.008 R^2^ gain that disappears entirely under scaffold splitting (*p* = 0.599) reflects scaffold co-occurrence artifacts rather than learnable geometric signals, a familiar overfitting pattern in which a marginal feature set captures scaffold-specific details rather than transferable physicochemical information.

This conclusion has a mechanistic basis. Passive BBB permeation is primarily governed by the thermodynamic cost of desolvating the polar molecular surface and the energetics of membrane partitioning. The TPSA descriptor approximates the former by summing atom-type-specific increments over the molecular graph. TPSA dominated the predictive models in the present study and in our previous investigation of the B3DB dataset. The finding that 3D PSA (PNSA1 from the CPSA family) correlates only moderately with 2D TPSA (r = 0.52) yet shows identical univariate log BB predictability (r^2^ ≈ 0.29 for both) is particularly informative: the 3D geometric detail diverges numerically but does not reduce the residual variance in the log BB. This suggests that polar atom topology, namely, which atoms are polar and how many are present, is more important for BBB permeation than the precise three-dimensional arrangement within a conformer. The examination of all four pre-specified pairwise comparisons involving 3D descriptors further supports the consistency of this conclusion. The 3D-only feature set underperformed the 2D-only representation by 0.137 R^2^ units under random splitting and 0.120 R^2^ units under scaffold splitting in the test set. Adding 3D descriptors to molecular fingerprints (FP + 3D vs. FP) resulted in a marginal improvement of +0.009 R^2^ under random splitting; however, this difference was not significant under scaffold splitting, showing a pattern consistent with the 2D + 3D versus 2D comparison. Similarly, adding 3D descriptors to the combined 2D + FP representation did not produce a significant performance change for either split type (*p* = 0.080 for random splitting; *p* = 0.801 for scaffold splitting). Overall, the consistent trend across all four comparisons indicates that the limited incremental contribution of the 3D descriptors is not dependent on a specific baseline representation or model configuration. A similar behavior was observed whether 3D features were evaluated independently, combined with 2D topology, combined with molecular fingerprints, or incorporated into the complete 2D + FP feature space.

The scaffold-split results highlight the most practically important validation lessons. The 2D descriptor model lost 0.149 R^2^ units from random to scaffold splitting, fingerprints lost 0.129 units, and 3D descriptors lost 0.132 units. None of the six feature sets achieved a scaffold-split R^2^ above 0.423, confirming that scaffold generalization, rather than feature representation, is the primary bottleneck in current BBB regression models. Improving scaffold generalization will require either architectural advances (e.g., graph neural networks with scaffold-aware message passing, Bayesian uncertainty quantification, and meta-learning) or substantial expansion of the training set with a balanced representation of uncommon scaffold classes rather than enriched molecular featurization. Several methodological choices require justification. First, using a single algorithm (LightGBM) for all 30-repeat comparisons ensures that paired statistical tests compared feature representations under identical algorithmic conditions, eliminating algorithm-selection variance as a confound. Second, using 30 independent replicates rather than the conventional 5–10 provides substantially tighter confidence intervals (mean SD across feature sets ≈0.06 random, ≈0.09 scaffold) and resolves the borderline effects at 5-repeat sample sizes that would have had insufficient power to distinguish them from noise. Third, by reporting both random and scaffold-based validation results while treating the scaffold-based *p*-value as the primary evidence of generalizability, we addressed the concern that statistical significance under random splitting may be inflated by scaffold overlap between the training and test sets.

A comparison with the previous study on this dataset provides additional context and clarifies the novelty of the present work [11]. In that study, 40 descriptors were selected through iterative manual curation, with lipophilicity-related descriptors (SLogP and ALogP), hydrogen-bond donor count, TPSA, and ring system descriptors among the most influential features. The authors reported a test R^2^ of 0.604 using a single, optimized random partition. In contrast, the present automated MI + RF composite pipeline also identified TopoPSA (NO) as the top-ranked descriptor, demonstrating consistent descriptor-level agreement between the two studies and reinforcing the importance of polar-surface-related properties in BBB permeability prediction. In addition, the automated selection strategy retained autocorrelation descriptors (ATSC and AATSC families) and π-system connectivity indices (piPC1 and piPC4), which were not included in the manually curated descriptor set [11]. The difference in the reported R^2^ values (0.604 in [9] versus 0.567 in the present study) should be interpreted in the context of the different validation designs. While optimized descriptor selection [11] and model performance for a specific train–test partition, the present study applied the same feature-selection and modeling workflow across six feature representations and 30 independent random and scaffold-based splits. Thus, the present approach prioritizes methodological reproducibility and a fair comparison of molecular representations over the optimization of a single dataset partition.

The objective of the current study was not to maximize predictive accuracy but to quantify the incremental contribution of conformer-derived molecular information using a controlled and reproducible evaluation framework. Furthermore, the present study extends previous applications of 3D descriptors in BBB modelling [9,10] by providing, to our knowledge, a systematic evaluation of explicit 3D conformer descriptors using scaffold-based validation on a large standardized experimental log BB dataset, together with paired statistical comparisons against corresponding 2D representations. The limitations of this study include the reliance on single-conformer geometry per molecule (lowest MMFF94 energy from ETKDGv3 embedding), the absence of WHIM, GETAWAY, and RDF descriptors due to Mordred implementation constraints, and the inherent limitation that log BB conflates passive permeability, active efflux, and tissue-binding mechanisms that individual descriptor classes cannot disentangle. Testing conformational ensembles and extended 3D descriptor libraries on a flexible subset of molecules would be a natural extension of this work.

## 5. Conclusions

This systematic evaluation across 30 independent random and scaffold-based splits on the B3DB benchmark demonstrates that three-dimensional conformer-derived descriptors do not provide scaffold-robust predictive improvements over optimized two-dimensional topological representations for BBB permeability prediction. Small numerical gains observed under random splitting (+0.008 R^2^ units for 2D + 3D vs. 2D; +0.007 for 2D + FP + 3D vs. 2D + FP) were not statistically significant under Murcko scaffold splitting (*p* = 0.599 and *p* = 0.801), indicating that these gains reflect scaffold co-occurrence rather than transferable geometry-dependent chemical properties of the compounds. The curated 2D descriptor model (LightGBM, 60 mordred descriptors) achieved consistent performance across split types (random: 0.567 ± 0.060; scaffold: 0.418 ± 0.085), with SHAP analysis confirming TopoPSA (NO) as the dominant predictor, consistent with the desolvation mechanism. The applicability domain analysis showed that 94.8% of the test compounds were within the structural coverage of the training set. These findings, obtained on the B3DB benchmark using Mordred-derived descriptors calculated from single ETKDGv3 + MMFF94 conformers, support the use of well-curated 2D descriptors as the default molecular representation for BBB regression tasks in virtual screening pipelines. Three-dimensional conformer generation should be considered for applications where molecular geometry is intrinsically required by the task design, such as molecular docking and structure-based virtual screening (where the three-dimensional ligand pose determines protein–ligand interactions), pharmacophore-based scaffold hopping (where the spatial arrangement of chemical features defines the pharmacophore hypothesis), and shape-based similarity searching. However, the present results do not demonstrate a consistent benefit of explicit 3D conformer descriptors for improving log BB regression accuracy within the ligand-based QSAR frameworks. Future studies should investigate whether alternative conformer ensembles, other 3D descriptor families such as WHIM and GETAWAY descriptors, or expanded training datasets alter these conclusions beyond the specific conditions evaluated in this study.

## Figures and Tables

**Table 1:** Mean test R^2^ values obtained using LightGBM across 30 independent random splits and 30 independent Murcko scaffold-based splits for six molecular feature representations. Values are reported as mean ± standard deviation. ΔR^2^ denotes the difference between the scaffold and random performances and represents the loss in predictive accuracy associated with scaffold-based generalization. The full split-by-split results are presented in Supplementary Table S3.

Feature Set	Random R^2^	Scaffold R^2^	Generalization Loss (ΔR^2^)
2D descriptors (60 curated)	0.567 ± 0.060	0.418 ± 0.085	−0.149
ECFP4 fingerprints (1024 bits)	0.441 ± 0.049	0.312 ± 0.084	−0.129
3D conformer descriptors (40)	0.430 ± 0.066	0.298 ± 0.097	−0.132
2D + 3D	0.575 ± 0.054	0.420 ± 0.092	−0.155
2D + fingerprints	0.572 ± 0.057	0.421 ± 0.086	−0.151
2D + fingerprints + 3D	0.579 ± 0.052	0.423 ± 0.096	−0.156

**Table 2:** Paired statistical comparisons of predictive performance across molecular feature representations were based on 30 independent replications. Results are reported for both the paired Student’s *t*-test and Wilcoxon signed-rank test under random and Murcko scaffold-based validations. Scaffold-based results were considered as primary evidence for evaluating the robustness and transferability of predictive improvements across unseen chemical scaffolds.

Comparison	Random *t*-Statistic	Random *p*-Value	Random Wilcoxon *p*-Value	Scaffold *t*-Statistic	Scaffold *p*-Value	Scaffold Wilcoxon *p*-Value	Interpretation
2D vs. 2D + 3D	−2.167	0.039	0.055	−0.532	0.599	0.477	Marginal improvement under random splitting; not significant under scaffold-based validation
2D + FP vs. 2D + FP + 3D	−1.816	0.080	0.205	−0.255	0.801	0.984	No significant difference
2D vs. FP-only	16.735	<0.001	<0.001	12.146	<0.001	<0.001	Two-dimensional descriptors significantly outperform fingerprints
2D vs. 2D + FP	−2.036	0.051	0.033	−1.078	0.290	0.393	Marginal improvement under random splitting; not significant under scaffold-based validation

## Data Availability

The B3DB dataset used in this study is publicly available in the B3DB repository. The processed dataset, curated descriptor set, split-by-split model performance results, statistical analyses, and SHAP rankings supporting the findings of this study are provided in the Supplementary Materials. Additional information is available from the corresponding author upon reasonable requests.

## Supplementary Materials

The following supporting information can be downloaded at: https://www.mdpi.com/article/10.3390/pharmaceutics18081018/s1. Table S1. B3DB regression dataset and 3D conformer status. Table S2. Sixty selected 2D Mordred descriptors. Table S3. LightGBM test R^2^ across six feature sets and 60 validation splits. Table S4. Statistical comparisons of feature-set performance. Table S5. Top 20 SHAP features of the 2D descriptor LightGBM model.
